# Gas Permeation Properties of Soluble Aromatic Polyimides Based on 4-Fluoro-4,4'-Diaminotriphenylmethane

**DOI:** 10.3390/ma8041951

**Published:** 2015-04-21

**Authors:** Diego Guzmán-Lucero, Jorge Froylán Palomeque-Santiago, Claudia Camacho-Zúñiga, Francisco Alberto Ruiz-Treviño, Javier Guzmán, Alberto Galicia-Aguilar, Carla Aguilar-Lugo

**Affiliations:** 1 Instituto Mexicano del Petróleo, Eje Central Lázaro Cárdenas Norte 152, 07730 México, Mexico; E-Mails: jpalomeq@imp.mx (J.F.P.-S.); jguzmanp@imp.mx (J.G.); 2 Universidad Iberoamericana, Departamento de Ingenierías. Prol. Paseo de la Reforma No. 880, Col. Lomas de Santa Fe, C. P. 01219 México, Mexico; E-Mails: P23941@correo.uia.mx (C.C.-Z.); alberto.ruiz@ibero.mx (F.A.R.-T.); 3 Facultad de Ingeniería Química, Benemérita Universidad Autónoma de Puebla, 14 sur y San Claudio, Ciudad Universitaria, 72592 Puebla, Pue., Mexico; E-Mail: albertogaliciaaguilar@yahoo.com; 4 Instituto de Investigaciones en Materiales, Universidad Nacional Autónoma de México, Circuito exterior s/n, Cuidad Universitaria, 70360 México, Mexico; E-Mail: carla.aguilar.lugo@gmail.com

**Keywords:** polyimides, 4-fluoro-4'-4"-diaminotriphenylmethane, gas separation membranes

## Abstract

A series of new organic polyimides were synthesized from 4-fluoro-4'4"-diaminotriphenylmethane and four different aromatic dianhydrides through a one-step, high-temperature, direct polycondensation in m-cresol at 180–200 °C, resulting in the formation of high-molecular-weight polyimides (inherent viscosities ~ 1.0–1.3 dL/g). All the resulting polyimides exhibited good thermal stability with initial decomposition temperatures above 434 °C, glass-transition temperatures between 285 and 316 °C, and good solubility in polar aprotic solvents. Wide-angle X-ray scattering data indicated that the polyimides were amorphous. Dense membranes were prepared by solution casting and solvent evaporation to evaluate their gas transport properties (permeability, diffusivity, and solubility coefficients) toward pure hydrogen, helium, oxygen, nitrogen, methane, and carbon dioxide gases. In general, the gas permeability was increased as both the fractional free volume and d-spacing were also increased. A good combination of permeability and selectivity was promoted efficiently by the bulky hexafluoroisopropylidene and 4-fluoro-phenyl groups introduced into the polyimides. The results indicate that the gas transport properties of these films depend on both the structure of the anhydride moiety, which controls the intrinsic intramolecular rigidity, and the 4-fluoro-phenyl pendant group, which disrupts the intermolecular packing.

## 1. Introduction

Polyimides (PIs) are considered high-performance polymers because of their excellent physical and chemical properties, including good chemical stability over a broad temperature range. The applications of PIs in advanced technologies have been the subject of considerable research since the development of soluble PIs, which has resulted in the improved processability of PIs [1]. Researchers have also focused their works on the use of PIs as gas separation membranes [2,3,4]. 

Gas separation using polymeric membranes has advantages over traditional technologies such as easy-to-operate compact equipment and lower energy requirements [5]. Their application as replacements for or in combination with such technologies in different separation and purification processes [6,7], including O_2_/N_2_ (e.g., nitrogen generation) and CO_2_/CH_4_ (e.g., natural gas separation), depends strongly on the properties of the membrane. In addition to being mechanically, chemically, and thermally stable, polymeric membranes should also exhibit high permeability and selectivity. The first property will increase the productivity of the process, whereas the second one will ensure the quality of the separation. Robeson [8] stated that these properties exhibit an inverse relationship. However, researchers have succeeded recently in producing materials, including some PIs [9] that overcome this inverse relationship by designing chemical structures that enhance the fractional free volume (FFV) [10,11,12,13,14].

Most aromatic PIs have strong interchain attractive forces, dense molecular packing, and stiff main chains, which create difficulties in polymer processing and poor solubility in common organic solvents. The introduction of bulky groups [15,16,17,18] and flexible linkages [11,19,20] into the polymer backbone represents several approaches to overcome such difficulties. However, the inclusion of bulky pendant groups increases the interchain spacing and reduces the packing efficiency, thereby increasing the FFV. Therefore, if the chemical structures are properly selected, these characteristics could avoid the permeability/selectivity tradeoff. 

Polyimides based on 4,4'-diaminotriphenylmethane (TM) have been previously synthesized using various methods. The well-known high-temperature one-step method has proven to be the most appropriate in terms of the thermal and mechanical properties of the products [21,22,23]. In this study, four new polyimide membranes based on 4-fluoro-4'4"-diaminotriphenylmethane (TMF) were studied with respect to their gas transport properties. Diaminotriphenylmethanes can be easily prepared from anilines and benzaldehydes, as reported elsewhere [23]. Here, dense membranes of aromatic polyimides from commercial dianhydrides and TMF were prepared; the polyimides were specifically designed and developed to be tested as gas separation membranes for various gases of commercial interest.

## 2. Experimental Section

### 2.1. Materials 

3,3',4,4'-Oxydiphthalic dianhydride (OD), 3,3',4,4'-benzophenone tetracarboxylic dianhydride (BT), 4,4',5,5'-sulfonyldiphthalic anhydride (DS), and 4,4'-(hexafluoroisopropylidene)diphthalic anhydride (6F) were purchased from Sigma-Aldrich Co. (St. Louis, MO, USA). Before use, these reagents were recrystallized from acetic anhydride and sublimed two times under reduced pressure. Aniline, N,N-dimethylformamide (DMF), 4-fluorobenzaldehyde, and *m*-cresol were purchased from the same supplier and used without further purification.

### 2.2. Polymer Synthesis

The 4-Fluoro-4,4'-diaminotriphenylmethane monomer was prepared from aniline and 4-fluorobenzaldehyde and purified as described in the literature [23].

**Figure 1:**
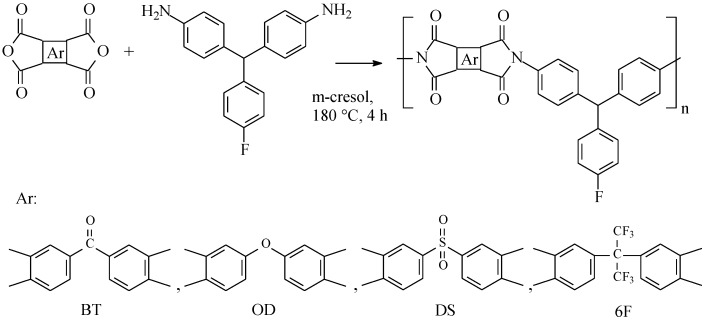
Chemical structures and reaction scheme for synthesizing the four polyimides reported in this work.

Polyimides were synthesized via a conventional one-step high-temperature polycondensation reaction, as shown in Figure 1. The resulting polymers were precipitated in methanol, dissolved in DMF, and reprecipitated three times in methanol to eliminate most of the m-cresol. The resulting PIs were then vacuum-dried at 120 °C for 10 h.

### 2.3. Measurements

The chemical structures of the obtained PIs were investigated using FT-IR spectroscopy, performed with a Bruker Equinox 55 spectrometer (Ballerica, MA, USA). Their thermal properties were determined by high-resolution simultaneous thermogravimetric analysis (TGA) and differential scanning calorimetry (DSC) performed using a Netzsch STA 409 (Selb, Germany); the samples were heated at 5 °C/min under a nitrogen atmosphere. The inherent viscosities (η_inh_, 0.5 g/dL in DMF solutions) were determined at 25 °C using an Ubbelohde viscometer and a Cannon CT-500 series II constant temperature bath (±0.01 °C). Wide-angle X-ray scattering (WAXS) measurements were performed on a Siemens D500 (Karlsruhe, Germany) X-ray diffractometer (graphite-monochromated, Ni-filtered Cu-Kα radiation). The polymer densities were measured, with an accuracy of 0.0001 g/cc, in a jacketed density gradient column filled with a well-degassed aqueous potassium iodide solution at 23.0 ± 0.1 °C. Each dried polymer sample was first wetted in the low-density solution and then transferred to the column. Two samples of each material were used to determine the density. Although they passed rapidly through the column, the measurements were performed after 12 h to ensure that they had reached equilibrium. The density values were used to calculate the FFV as follows:

(1) $$FFV=\frac{V-V_{0}}{V}$$

where *V* is the specific volume; and *V*_0_ is the occupied volume of the polymer calculated from:

(2) $$V_{0}=1.3\sum_{i=1}^{n}V_{wi}$$

where *V_w_* corresponds to the Van der Waals volume of each group that makes up the polymer repeating unit according to Bondi’s group contribution method [24,25].

### 2.4. Preparation of Dense Membranes

Polymer dense films were produced on laboratory scale by casting a polymer solution (15%–20%, w/v) in DMF onto glass plates at room temperature using a casting blade. The removal of solvent was achieved by controlling the pressure and temperature. The polymer films were initially vacuum-dried at 40 °C for 4 h; subsequently, the temperature was increased to 120 °C for an additional 10-h period. The membranes were detached from the glass substrate and mounted on steel frames, where they were maintained at 250 °C for an additional 8-h period under a vacuum. The complete removal of solvents was confirmed by TGA. All membranes were treated by using the same procedure, and their thicknesses ranged between 40 and 70 µm, as measured using a Mitutoyo digital micrometer with an accuracy of ±1 µm.

### 2.5. Gas Permeability Measurements

Pure gas permeability coefficients, at 35 °C and 2 atm upstream pressure, for the PIs synthesized in this work were measured in a standard constant-volume, variable-pressure permeation cell. Six gases were tested in the following order to avoid plasticization or conditioning of the membrane: hydrogen, helium, nitrogen, oxygen, methane, and carbon dioxide. The gas permeability coefficients were determined from the slope of the downstream pressure *vs.* time plot after a steady state had been achieved. The gas diffusivity coefficients for each gas (*D*) were estimated from the time-lag data (θ) using the following equation:

(3) $$D=\frac{l^{2}}{2\text{θ}}$$

where *l* is the film thickness.

The thermodynamic solubility coefficients for each gas (*S*) were obtained from the expression:

(4) $$S=\frac{P}{D}$$

where *P* is the permeability coefficient for each gas. The ideal selectivity values (α_A/B_) between gases *A* and *B* were calculated from the pure permeability coefficients as follows:

(5) $${\text{α}}_{A/B}=\frac{P_{A}}{P_{B}}$$

## 3. Results and Discussion

After optimization of the synthesis conditions, high-molecular-weight polyimides were synthesized through the one-step polycondensation method in *m*-cresol at 180–200 °C. The reaction solutions with a concentration of solids between 10 and 15 wt% were vigorously stirred for 4 h under flowing nitrogen. All monomers used in this study were dissolved completely in the reaction solutions under these conditions, and the resulting polymers did not precipitate, even when cooled to room temperature. 

**Figure 2:**
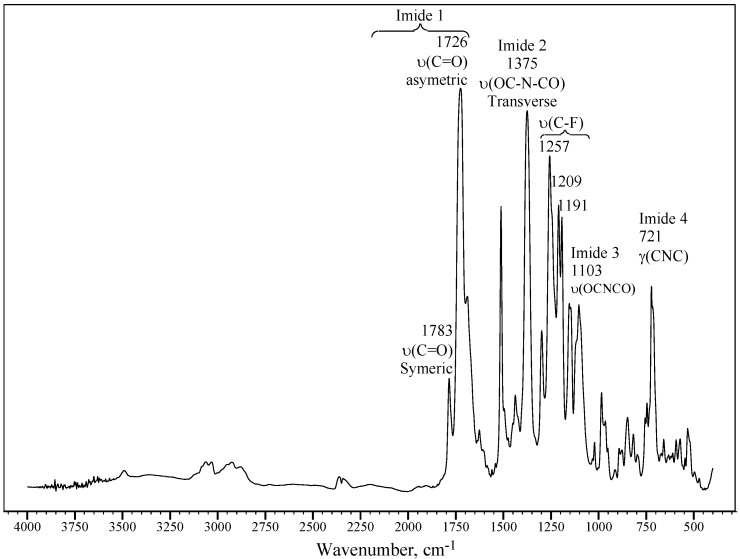
FTIR spectra determined for a 6F-TMF polyimide synthesized in this work.

The chemical structures of the polyimides used in this study are shown in Figure 1. It can be seen that these polyimides are distinguished by the inclusion of the bulky pendant 4-fluoro-phenyl group in their diamine moiety and their chemical structures were studied by FTIR spectroscopy. As an example, Figure 2 presents the FTIR spectrum of 6F-TMF. The characteristic absorptions of the imide group at approximately 1778 and 1724 cm^−1^ (imide 1 ν(C=O)), 1373 cm^−1^ (imide 2 ν(OC–N–CO)), 1091 cm^−1^ (imide 3 ν(OC–N–CO)) and 721 cm^−1^ (imide 4 γ (CNC)) were identified. Strong multiple vibration peaks of ν(C–F) at 1257, 1209, and 1191 cm^−1^ of the hexafluoroisopropylidene group were also observed for the 6F-TMF polyimide. In addition, a peak at approximately 1209 cm^−1^, corresponding to the ν(C–F) linkage present in the phenyl rings of OD-TMF, BT-TMF, and DS-TMF, was observed. All these absorption signals clearly indicate the presence of imide groups [26] and confirm the successful synthesis of the desired structures.

The solubility of the polyimides was determined at room temperature and at a 5 wt% concentration in some organic solvents, as shown in Table 1. All the PIs exhibited good solubility at room temperature in solvents such as DMF, *N*-methyl-2-pyrrolidone, *N*,*N*-dimethylacetamide, nitrobenzene, and *m*-cresol, and partial solubility in CHCl_3_ was displayed by the BT-TMF, OD-TMF, and DS-TMF polyimides, except the 6F-TMF PI, which was soluble. Such behavior is related to the presence of the 4-fluoro-phenyl pendant group. The bulkiness and free internal rotation of this pendant group disrupts the molecular packing, thereby easing the penetration of solvent molecules among the polymer chains to dissolve it.

**Table 1:** Summary of solubility tests carried out with 5 wt% of TMF-based polyimides dissolved in several organic solvents.

Polymer	Solvent							
	CHCl_3_	DMF	DMSO	NMP	Nitro-Bz	*m*-Cresol	DMAc	THF
OD-TMF	±	+	+	+	+	+	+	−
BT-TMF	±	+	+	+	+	+	+	−
DS-TMF	±	+	+	+	+	+	+	−
6F-TMF	+	+	+	+	+	+	+	±

+ Soluble, ± Partially soluble, − Insoluble: DMF: N,N-dimethyl formamide DMSO: dimethyl sulfoxide; Nitro-Bz: Nitrobenzene, THF: tetrahydrofuran, DMAc: N,N-dimethyl acetamide.

**Figure 3:**
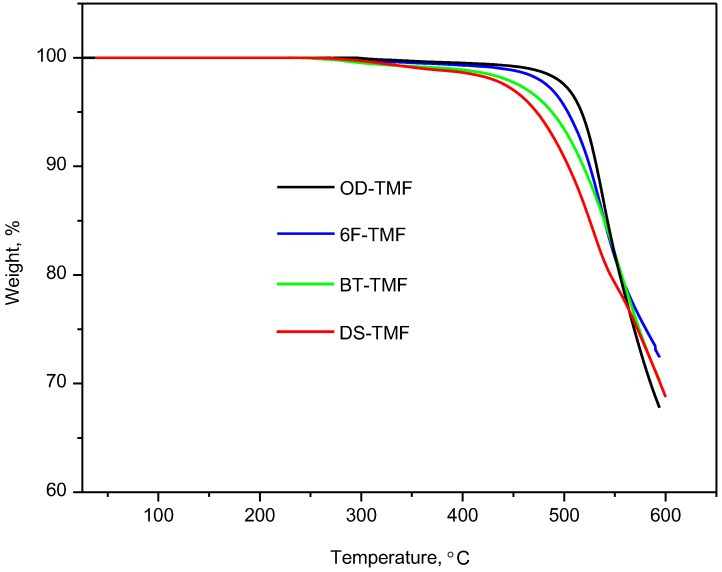
TGA curves measured under N_2_ atmosphere for the TMF-based polyimides reported in this work.

The thermogravimetric analyses determined under a nitrogen atmosphere for these TMF-based polyimides are shown in Figure 3. These PIs exhibit good thermal stability and have degradation temperatures, for a 5 wt% mass loss, between 472 °C for DS-TMF and 517 °C for OD-TMF; their residual weight at 600 °C is above 68% (Table 2).

**Table 2:** Some important physical properties determined for the TMF-based polyimides synthesized in this work.

Polyimide	5% weight loss, °C	Residual weight at 600 °C, %	η_inh_, dL/g	T_g_, °C	Density, g/cm^3^	*d*-Spacing, Å	FFV
OD-TMF	517	68	1.2	285	1.328	4.8	0.173
BT-TMF	487	70	1.3	290	1.331	5.0	0.178
DS-TMF	472	69	1.0	316	1.354	5.2	0.184
6F-TMF	503	72	1.2	297	1.379	6.8	0.205

**Figure 4:**
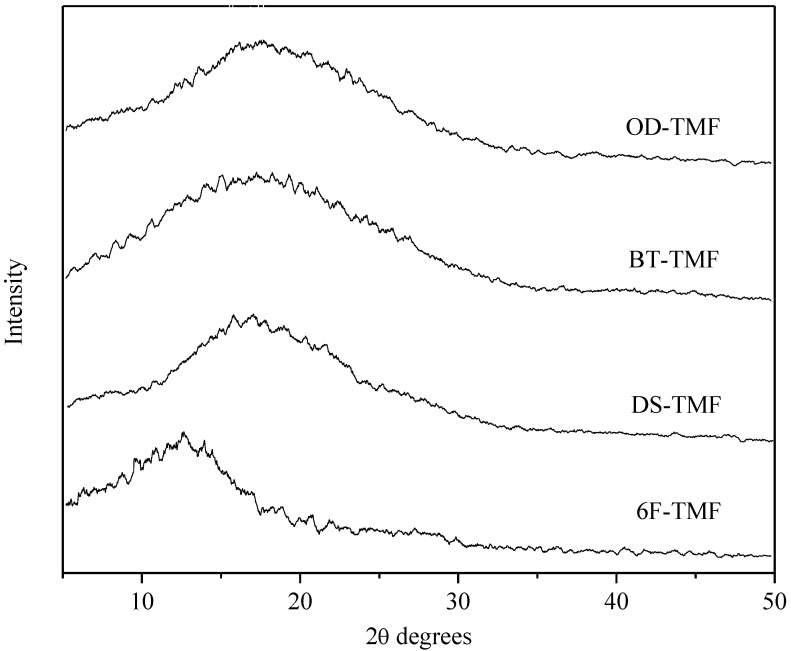
WAXD patterns determined for TMF-based polyimide membranes.

Figure 4 presents wide-angle X-ray diffractograms determined for the polyimide membranes synthesized in this work. The diffractograms are broad and structureless, which indicates that these PIs are amorphous, an important requirement for highly productive gas separation membranes [27]. The amorphous nature of these polyimides can be attributed, in part, to the presence of the bulky 4-fluoro-phenyl pendant group in the diamine moiety, which leads to a loosely packed structure. 

Table 2 summarizes the decomposition temperature (for a 5 wt% polymer loss), the residual weight at 600 °C, the inherent viscosity for polymer solutions, the glass transition temperature, and the density measured for the PIs synthesized in this work, as well as the corresponding *d*-spacing, calculated using the Bragg’s equation applied to the most prominent peak shown in the WAXS diffractograms (see Figure 4) and also the corresponding FFV calculated from Equations (1) and (2). The inherent viscosity values between 1.0 and 1.3 dL/g confirm that these polymers possess reasonably high molecular weights, leading to the formation of flexible membranes with sufficient mechanical strength for gas permeation measurements. All the PIs have high glass-transition temperature (T_g_) values that range from 285 to 316 °C. An increase in T*_g_* generally corresponded to an increase in rigidity of the dianhydride monomer. Bridging groups, such as –O– and –C=O (OD-TMF and BT-TMF polyimides), between the two phenyl rings present in the dianhydrides clearly facilitate bond rotation and consequently reduce the T_g_ [15]. In contrast, 6F-TMF and DS-TMF with –C(CF_3_)_2_ and –S(O)_2_– connectors have higher T*_g_* values because molecular motions are inhibited. The changes in *d*-spacing provide an indicator of the amount of available space for small molecules to penetrate, and it is observed that except for the 6F-TMF polyimide, where the *d*-spacing is relatively higher than the other three PIs, the *d*-spacing values were not changed significantly as a consequence of the chemical changes introduced in the polymer repeating unit. The *d*-spacing for OD-TMF, BT-TMF and DS-TMF are practically the same, from 4.8 to 5.2 Å, whereas for 6F-TMF, the calculated d-spacing is in the order of 6.8 Å. The restricted torsional motion around a –C(CF_3_)_2_ linkage suppressed efficient polymer chain packaging and this has to be reflected in the permeability and selectivity combination of properties for membranes based on 6F-TMF polyimide. It is important to mention that the fluorinated polyimides synthesized in this work have a *d*-spacing that is higher than those featured by other polyimides reported elsewhere [15,19,28,29,30,31,32,33]. This illustrates the merit of introducing the 4-fluoro-phenyl pendant group into the polymer backbone for inhibiting chain packaging. With respect to the FFVs shown in Table 2, the values of TMF-based polyimides are ranked in the same order as the density values: OD-TMF < BT-TMF < DS-TMF < 6F-TMF. The highest FFV value = 0.205 in 6F-TMF can be explained by the significantly higher weight of the –C(CF_3_)_2_ linkage and its bulkiness, which increases chain stiffness and decreases the packing ability. In fact,–C(CF_3_)_2_ linkages in 6F-based polyimides are known to favor drastically solubility [30], increase the free volume, and decrease the intermolecular interactions between the polymer chains [34]. A last observation in Table 2 is related to the excellent agreement observed between *d*-spacing and FFV. In general, polymers with larger *d*-spacing tend to have larger FFV [19], and this generality is also valid for this series of polyimides.

Table 3 reports the permeability coefficients measured for the synthesized PIs to H_2_, He, O_2_, N_2_, CH_4_, and CO_2_, as well as the ideal selectivity to several gas pairs. Note that they rank in the following order: OD-TMF < DS-TMF ~ BT-TMF < 6F-TMF. This trend is very similar to that of the *d*-spacing and FFV, considering that the differences between the permeability coefficients of BT-TMF and DS-TMF are below 6.3%, a percentage comparable to the measurement error. It is evident that the dianhydride moiety in the structure of the TMF-based polyimides exerts a strong influence on both the observed permeability and the permeability sequence for small molecular gases. Notably, the permeability coefficients of 6F-TMF are greater than those featured by other PIs by one order of magnitude, e.g., P(H_2_) = 60 Barrers, P(O_2_) = 6.8 Barrers and P(CO_2_) = 35 Barrers. The introduction of the 4-fluoro-phenyl pendant group into the polymer backbone induced a significant permeability improvement when these values are compared to the permeability coefficients of similar polymers reported elsewhere [15,19,28,29,30,31,32,33]. For example, the CO_2_ permeability coefficient of 6F-TMF is either 3 times higher than those of 6F-HAB [29] and 6F-biphenyl [32] or 1.5 times higher than that of 6F-MDA [33]. The substitution of the bulkier 4-fluoro-phenyl central group in 6F-TMF for either –CH_2_– (6F-MDA) or –O–Ph(Ph)–O–(6F-biphenyl) or introduction between aromatic rings in 6F-HAB leads to simultaneous disruption in intermolecular packaging and suppression of intrarotational flexibility in the diamine segment of these polyimides. Moreover, in comparison with some permeability results reported in the literature, PIs derived from BT-, OD-, and DS-dianhydrides and TMF-based PIs show relatively higher or comparable permeability. For example, the CO_2_ permeability of the BT-TMF membrane is higher than that of BT-pp’ODA [30] (0.62 Barrers) or BT-ODA [31] (0.63 Barrers), and comparable to that of BT-DATPA [31] (3.3 Barrers), derived from the same BT dianhydride. Even for smaller gas molecules like He and H_2_, the permeability coefficients of TMF-based polyimides are higher than all of the 6F-based PIs, and also including BT-, OD-, and DS-based PIs such as OD-PDAB [15] (6.1 Barrers) and BT-ODA [31] (4.8 Barrers).

**Table 3:** Gas transport properties measured at 35 °C and 2 atm for the TMF-based polyimides reported in this work.

Polyimide	H_2_	He	Permeability *, P(A)				Ideal selectivity, P(A)/P(B)			
			O_2_	CO_2_	N_2_	CH_4_	H_2_/CH_4_	He/N_2_	O_2_/N_2_	CO_2_/CH_4_
OD-TMF	8.2	8.8	0.57	2.5	0.10	0.10	85	86	5.5	26
BT-TMF	9.6	10	0.73	3.2	0.14	0.12	78	72	5.2	26
DS-TMF	9.2	9.8	0.67	3.4	0.12	0.13	86	82	5.6	31
6F-TMF	60	58	6.8	35	1.30	0.85	70	45	5.3	41

* Permeability in Barrers with an associated uncertainly of 4%; 1 Barrer= 10^−10^ cm^3^(STP)cm/cm^2^ sec cmHg.

With respect to the ideal selectivity for the gas pairs reported in Table 3, it should be noted that the highest values measured for these PIs are 86 for H_2_/CH_4_ (DS-TMF), 86 for He/N_2_ (OD-TMF), 5.6 for O_2_/N_2_ (DS-TMF), and 41 for CO_2_/CH_4_ (6F-TMF). The introduction of the 4-fluoro-phenyl pendant group into the polymer backbone, which inhibits the molecular motion and packaging, enables the TMF-based PIs, in general, to have an increase in gas permeability with negligible decrease, or in the best case, with simultaneous increases in permeability and ideal selectivity as the 6F-TMF membrane shows for the pair of gases CO_2_/CH_4_. It is important to mention that the 6F-TMF membrane features the highest *d-spacing* and FFV as compared to the other three PIs and from the typical trade-off rules, this membrane would be expected to possess a higher permeability but lower selectivity than the other three PIs. However, this membrane shows the typical behavior observed in the polysulfone [35] and polycarbonate [36] families, where a replacement of the –C(CH_3_) group of either tetramethyl or tetrabromobisphenol A by the –C(CF_3_) group leads to simultaneous increases in *d-*pacing, FFV, CO_2_ permeability and CO_2_/CH_4_ ideal selectivity. As was mentioned before, the presence of –C(CF_3_) in these PI families improves significantly both the CO_2_ diffusivity and solubility with respect to the corresponding CH_4_ values. In general, the ideal selectivity measured for the four PIs studied in this work falls within the range of other PIs reported in current literature. For example, the CO_2_/CH_4_ selectivity of 6F-based polyimides such as 6F-TBAPB (26) [28], 6F-PDAB (37), 6F-BATPHF (32) [30] and 6F-DATPA (34) [31] are lower than that of 6F-TMF (41). In the case of the O_2_/N_2_ ideal selectivity, the results are comparable with those reported elsewhere [15,19,28,29,30,31,32,33].

**Figure 5:**
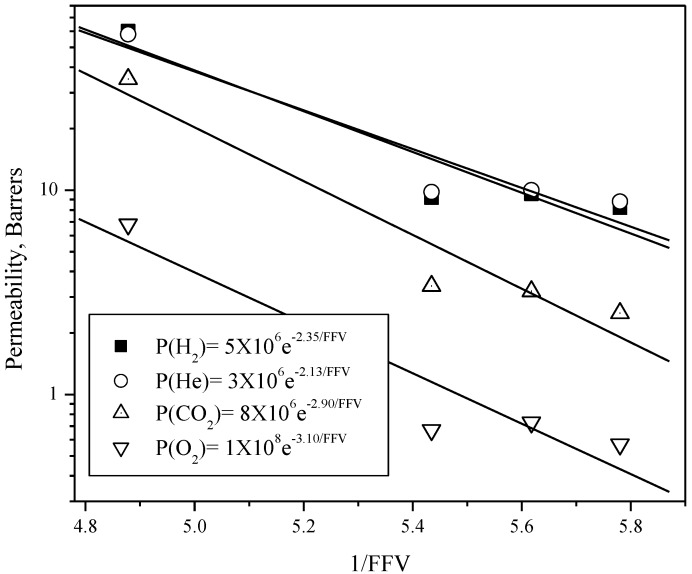
Gas permeability and 1/FFV relationship shown by the TMF-based polyimides.

In addition to the simultaneous increase in FFV and the permeability characteristics of amorphous polymers, the permeability coefficients correlate well with the inverse of the FFV according to the empirical equation:

(6) $$P=Ae^{-B/FFV}$$

where *A* and *B* are characteristic parameters for each gas, which may also depend to some degree on the polymer family [37]. Figure 5 shows a plot of these relationships and the trend-line equations for each gas.

Table 4 presents the gas diffusivity and solubility coefficients and their corresponding selectivity values. For each analyzed polymeric membrane, the diffusivity values are decreased as the kinetic diameter of the penetrating gas is increased. The permeability exhibits the same trend, indicating clearly that the permeation process is governed by kinetic factors. Moreover, the solubility coefficients are increased as the critical temperatures of the penetrant are also increased (T_N__2_ = 126.2 K > T_O__2_ = 154.6 K > T_CH__4_ = 190.5 K > T_CO__2_ = 304.1 K).

**Table 4:** Gas diffusivity and solubility coefficients calculated from Equations (3) and (4) using the permeability coefficients measured at 35 °C and 2 atm for the TMF-based polyimide membranes. Their contribution to the overall selectivity factors is also included.

Polyimide	Diffusivity × 10^8^, cm^2^/s				Diffusivity selectivity	
	D(N_2_)	D(O_2_)	D(CH_4_)	D(CO_2_)	D(O_2_)/D(N_2_)	D(CO_2_)/D(CH_4_)
OD-TMF	0.3	1.3	0.04	0.23	5.2	5.3
BT-TMF	0.4	1.1	0.05	0.22	2.4	4.4
DS-TMF	0.2	0.9	0.04	0.21	5.0	5.9
6F-TMF	1.2	4.7	0.19	1.53	3.6	7.9
**Polyimide**	**Solubility, cm^3^ (STP)/cm^3^ atm**				**Solubility selectivity**	
	**S(N_2_)**	**S(O_2_)**	**S(CH_4_)**	**S(CO_2_)**	**S(O_2_)/S(N_2_)**	**S(CO_2_)/S(CH_4_)**
OD-TMF	0.3	0.3	1.7	8.3	1.0	4.8
BT-TMF	0.3	0.5	1.9	10.9	2.2	5.8
DS-TMF	0.5	0.5	2.2	11.9	1.1	5.3
6F-TMF	0.8	1.1	3.4	17.4	1.3	5.2

A comparison of 6F-TMF with the other three TMF-based polymers reveals that its diffusivity coefficients to N_2_ (1.2 × 10^−8^ cm^2^/s), O_2_ (4.7 × 10^−8^ cm^2^/s), CH_4_ (0.19 × 10^−8^ cm^2^/s), and CO_2_ (1.53 × 10^−8^ cm^2^/s) are the largest, which is in agreement with its possessing the largest *d*-spacing (6.8 Å) and FFV (0.205) (see Table 2). The solubility coefficients of 6F-TMF for all the analyzed gases are also the largest as compared to the other three PIs, and this result might be related to the changes caused by the presence of the hexafluoroisopropylidene group. In fact, such increases in the diffusivity and solubility of permeants have been previously observed in other fluorinated amorphous polymers [30,38,39,40].

With respect to the contributions to the separation factor, it is observed that for the pairs of gases with significant differences in their kinetic radii, the diffusivity selectivity is larger than the solubility selectivity. On the contrary, both contributions are comparable for the pairs of gases with similar kinetic radii.

**Figure 6:**
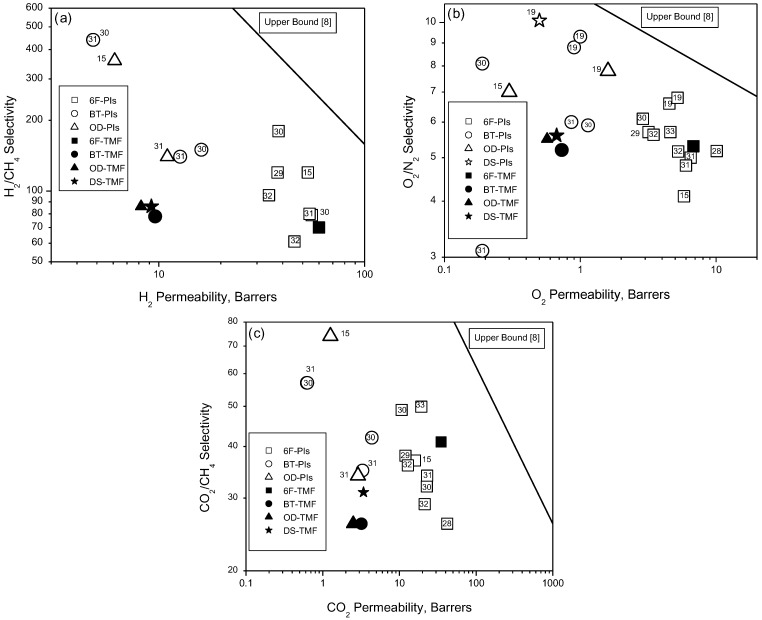
Selectivity and gas permeability combination of properties measured for the membranes based on the polyimides synthesized in this work: H_2_/CH_4_ (**a**); O_2_/N_2_ (**b**); and CO_2_/CH_4_ (**c**). Black or closed symbols correspond to PI membranes synthesized in this work, whereas open symbols are values reported elsewhere.

The selectivity–permeability relationships for the PIs studied in this work are shown in Figure 6 as updated 2008 Robeson’s upper bound [8] plots for the (a) H_2_/CH_4_; (b) O_2_/N_2_; and (c) CO_2_/CH_4_ gas pairs, in comparison with other reported polyimides [15,29,30,31,32,33] prepared from OD, BT, DS and 6F dianhydride. Notably, in the three analyzed pair of gases, the properties of OD-TMF, BT-TMF, and DS-TMF are quite similar. The differences among them are very subtle; consequently, properties other than gas transport must be considered as discriminants for any specific gas separation application.

For the H_2_/CH_4_ pair, Figure 6a demonstrates that from the four synthesized polymeric materials only the 6F-TMF membrane exhibits separation properties that are better than those displayed by 6F-terphenyl [32] and 6F-biphenyl [32]. For the O_2_/N_2_ pair, Figure 6b shows that the trade-off for the OD-TMF, BT-TMF, and DS-TMF polyimide membranes presents a permeability/selectivity ratio that is smaller than that for PIs reported in the literature [15,19,30,31]. Again, 6F-TMF presents the best selectivity/permeability data with respect to those displayed by the 6F-PDAB [15], 6F-HAB [29], 6F-DATPA [31], 6F-terphenyl [32], 6F-biphenyl [32], and 6F-MDA [33] polyimides.

For the CO_2_/CH_4_ gas pair, Figure 6c shows that 6F-TMF has the best performance regarding selectivity/permeability trade-off data with respect to those featured by 6F-PDAB [15], 6F-TBAPB [28], 6F-HAB [29], 6F-APAP [30], 6F-BATPHF [30], 6F-DATPA [31], 6F-terphenyl [32], and 6F-biphenyl [32]. The BT-TMF, OD-TMF, and DS-TMF polyimides have either equivalent or lower performances in terms of selectivity/permeability than those reported in the literature [15,19,30,31]. This leads to the conclusion, based on the limited results, that structural modifications that exclusively modify mobility factors are not sufficient for avoiding the well-known selectivity/permeability trade-off process.

## 4. Conclusions

Four new soluble polyimides containing 4-fluoro-4'-4"-diaminotriphenylmethane and BT, OD, DS, and 6F commercial dianhydrides were synthesized and characterized with respect to their thermophysical properties to explore their performance as gas separation membranes. High thermal decomposition temperatures (472–517 °C) and high glass-transition temperatures (285–316 °C) were exhibited by the polymers. The polyimide membranes were cast from their N,N-dimethylformamide solutions and resulted in transparent, flexible, and amorphous films, as indicated by X-ray diffraction measurements. 6F-TMF exhibited the highest *d*-spacing, density, free volume, and permeability because of the presence of the bulky hexafluoroisopropylidene linkage in the dianhydride moiety, and it also exhibited the best selectivity/permeability balance. Analysis of the structure/permeability relationship suggests that, when structural modifications only affect the contributions of mobility to gas transport, the solubility/permeability relationship corresponds to the well-known trade-off process. 
